# Multilingual Language Diversity Protects Native Language Production under Different Control Demands

**DOI:** 10.3390/brainsci13111587

**Published:** 2023-11-13

**Authors:** Keyi Kang, Yumeng Xiao, Hanxiang Yu, Michele T. Diaz, Haoyun Zhang

**Affiliations:** 1 Centre for Cognitive and Brain Sciences, University of Macau, Taipa, Macau SAR, China; 2 Department of Psychology, University of Macau, Taipa, Macau SAR, China; 3 Department of Psychology, The Pennsylvania State University, State College, PA 16801, USA

**Keywords:** multilingualism, language entropy, native language production, control demands, fMRI

## Abstract

The use of multiple languages has been found to influence individuals’ cognitive abilities. Although some studies have also investigated the effect of multilingualism on non-native language proficiency, fewer studies have focused on how multilingual experience affects native language production. This study investigated the effect of multilingualism on native language production, specifically examining control demands through a semantic Go/No-Go picture naming task. The multilingual experience was quantified using language entropy, which measures the uncertainty and diversity of language use. Control demands were achieved by manipulating the proportion of Go (i.e., naming) trials in different conditions. Results showed that as control demands increased, multilingual individuals exhibited poorer behavioral performance and greater brain activation throughout the brain. Moreover, more diverse language use was associated with higher accuracy in naming and more interconnected brain networks with greater involvement of domain-general neural resources and less domain-specific neural resources. Notably, the varied and balanced use of multiple languages enabled multilingual individuals to respond more efficiently to increased task demands during native language production.

## 1. Introduction

The ability to speak multiple languages is a common occurrence in today’s world, as globalization and multiculturalism continue to shape our societies. Many individuals are exposed to and use multiple languages in their daily lives, whether through immigration, education, or cultural immersion. Multilingualism has been extensively studied in psycholinguistic research as researchers seek to understand the cognitive processes underlying second language acquisition, bilingual language control, and the cognitive consequences of speaking multiple languages [1,2,3,4,5,6]. Yet, few studies have explored how the experience of using multiple languages, particularly the diversity (i.e., number of languages used) and uncertainty (i.e., probability of using a particular language in a specific language context), affects individuals’ ability to produce their native language. Moreover, although multilingualism has been shown to influence cognitive control [7,8], less is known about how the use of multiple languages interacts with these cognitive consequences during language production. The current study, therefore, investigates how multilingual experience modulates native language production in the context of manipulating control demands.

Language production often engages a left-lateralized frontal-temporal-parietal network [9,10]. Fluent production often involves cognitive control, as demonstrated in both behavioral and neural studies [11,12,13,14,15]. In contrast to monolinguals, multilinguals are challenged with processing information across all the languages they are proficient in and switching between languages to maintain effective communication in various contexts. Several studies have suggested that bilinguals’ language production places greater demands on competition and cognitive control than monolingual individuals [16,17,18]. The constant need to control two languages has led to research focusing on the cognitive consequences of lifelong bilingualism. Many studies have reported that bilinguals have cognitive and neural benefits compared with monolinguals [7,8], especially under higher task demands [19,20,21]. A common distributed network including the bilateral inferior frontal and temporal cortices and the anterior cingulate gyrus was found to be more efficiently activated in bilinguals across different cognitive control tasks [22,23,24]. Moreover, how multiple languages are used in daily communicative contexts is expected to modulate the effect of multilingualism on cognitive and brain functions [25,26].

While there is growing evidence of non-language cognitive benefits associated with using multiple languages [8,27,28], fewer studies have asked how this experience affects language processing. In addition to non-language cognitive benefits, some studies have also reported that multilingual experience has certain benefits for general language ability (e.g., metalinguistic awareness, effective selection of the language to be spoken, and linguistic reasoning) [29,30,31]. Yet, other studies have disclosed certain disadvantages in language processing for multilinguals compared with monolinguals. For instance, the Frequency Lag Hypothesis suggests that bilingual performance in both languages is worse than that of monolingual speakers due to their less frequent exposure to each language [32]. In addition, the Dynamic Model of Multilingualism [33] pointed out that individuals who need to manage two or more language systems may experience a heavier language load, potentially leading to disadvantages in language ability. These hypotheses have been supported by various findings showing that, compared with their monolingual peers, bilinguals have smaller vocabularies [34], lower scores on verbal fluency [35], and more tip of the tongue experiences [36].

One of the starkest consequences reflecting the potential downsides of multilinguals’ language ability is known as first language (L1) attrition, which refers to the gradual loss of proficiency or fluency in an individual’s first language resulting from exposure to other languages [37,38]. L1 attrition has often been found in the immigration population because of the change in the language environment. The loss of L1 is also related to the age of onset of bilingualism, the influence of L2 exposure and proficiency, as well as insufficient activation of L1 [39,40,41,42,43]. All these factors highlight the necessity of considering language usage and exposure comprehensively [44]. Yet, it is uncertain if multilingual individuals who still regularly use their native languages while also speaking other languages are susceptible to the same language attrition as those who undergo a change in the language environment. Alternatively, could their balanced and diverse language experiences potentially enhance their native language abilities? Some previous studies have found that multilinguals who live in their L1 environments still encounter difficulties in sentence parsing, processing of morphosyntactic variation, and comprehending or expressing complex ideas in their first language [45,46,47]. Regarding L1 production, Parker Jones and Green [48] found that bilinguals need to recruit more brain resources to achieve the same level of performance as monolinguals. On the other hand, Rossi and Newman [49] found that, with greater brain activation, bilinguals had higher accuracy in picture naming than monolinguals. Furthermore, while studies have revealed that highly proficient bilingual individuals tend to engage language-related and domain-general cognitive areas more extensively than monolinguals during language production [48,49,50,51], the functional neuroanatomic underpinnings of native language processing in multilingual individuals with more diverse language experiences remain unidentified.

As touched upon briefly, research on the effects of multilingualism should emphasize a comprehensive understanding of the multilingual experience, regardless of whether the effect is on cognitive control or language processing. In recent years, research on multilingualism has moved away from treating bilingualism or multilingualism as a categorical variable. Instead, researchers have begun to recognize the heterogeneity of language use by taking a more context-specific approach [25,52,53,54,55,56,57]. This more nuanced approach acknowledges the way in which languages are used, the frequency of use, and the level of proficiency can vary widely among multilingual individuals, and each dimension can significantly influence cognitive and language abilities [58,59,60,61].

To estimate individual- and contextual-level differences in language engagement, a measurement called language entropy has been developed [53]. Specifically, language entropy measures the degree of uncertainty and diversity when using multiple languages in different contexts, with high entropy indicating equal engagement and less predictable use of different languages and low entropy indicating a predictable use of one dominant language. This measurement can facilitate our understanding of the extent of multilingual usage and its impact on cognitive control and language processing. Using language entropy, some studies have reported that higher entropy values correlate with enhanced subjective and objective assessments of second-language proficiency [52], along with increased proactive control engagement [62,63]. From a functional connectivity standpoint, individuals with higher language entropy were found to exhibit increased brain network specialization and segregation, primarily in the default mode and executive control networks, as well as stronger connectivity between the anterior cingulate cortex and bilateral putamen [62,64]. Yet, some studies have reported that higher entropy is associated with smaller vocabulary in the second language [65]. Despite these findings, few studies have asked how language entropy would modulate native language ability.

In summary, despite numerous studies investigating the relationship between multilingualism and cognitive control, few have examined the impact of multilingual experience on native language production. Therefore, in the current study, we investigated how the experience of using multiple languages affects both behavioral and neural mechanisms of native language production, focusing on not only functional activation but also task-based functional connectivity. Recently developed measures, such as language entropy, have enabled researchers to capture the diversity in multiple language use more accurately. If higher language entropy is associated with worse production performance and less efficient neural processing, it would provide evidence suggesting that the diverse multilingual experience impairs L1 performance. Conversely, if higher language entropy is associated with enhanced L1 production and more efficient neural processing, it would suggest that the extensive use of multiple languages benefits L1 processing. Additionally, previous studies have suggested that control demands affect language production performance and result in greater activation in frontal-parietal-temporal circuits [66,67,68]. Furthermore, the effect of multilingualism on language and control processes might be modulated by task difficulty during production [19,20,21]. Therefore, we manipulated the control demands (i.e., task difficulty) involved in language production using a Go/No-Go picture naming paradigm, which has been validated in previous studies [69,70]. If the mixed and frequent use of multiple languages is beneficial, particularly in dealing with more challenging tasks, we would expect to observe a larger difference between high- and low-entropy individuals when the control demands are higher. Last but not the least, the participants included in the study were all multilinguals, speaking at least three languages, and had extensive experience using multiple languages from childhood. Therefore, the current study highlights a uniquely rich multilingual context where many different languages are being used.

## 2. Methods

### 2.1. Participants

Forty healthy participants were originally recruited, and four were excluded from the analysis: one for having excessive head movement (greater than 1/2 voxel), one for misunderstanding the instructions, and two others whose accuracy for the fMRI task was lower than 65%. The remaining 36 participants (27 females; aged 18–26 years, mean age = 21.1 years, SD = 1.8 years) were all native Cantonese multilinguals who were also highly proficient in Mandarin Chinese and had experience of learning and speaking another language, such as English, Portuguese, or Japanese. To understand language proficiency and overall multilingual language diversity, all participants were asked to complete a Language History Questionnaire (LHQ3) [71]. The overall proficiency of each language, ranging from 0 to 1, was computed based on the average score of the participant’s self-rated proficiency on different components (i.e., speaking, listening, reading, and writing) of each of their languages. Paired-sample *t*-tests showed that all participants were significantly more proficient in Cantonese compared with Mandarin Chinese (Cantonese: mean = 0.85, SD = 0.13; Mandarin Chinese: mean = 0.79, SD = 0.15; *t* = 2.77, *p* < 0.01), and both Cantonese and Mandarin Chinese proficiency were significantly higher than other languages (other languages: mean = 0.58, SD = 0.10; Cantonese vs. other languages: *t* = 10.28, *p* < 0.001; Mandarin Chinese vs. other languages: *t* = 7.27, *p* < 0.001).

All participants were right-handed, reported no history of psychological or neurological disorders, and had normal or corrected-to-normal vision. Before the MRI session, all participants completed a behavioral neuropsychological testing session to gauge their language and cognitive abilities. The neuropsychological tests utilized in this study and the assessment scores are reported in Table 1. Participants all signed consent prior to the beginning of the study. The study protocols, procedures, and consent forms were approved by the Research Ethics Committee of the University of Macau.

### 2.2. Stimuli and Procedure

**Figure 1:**
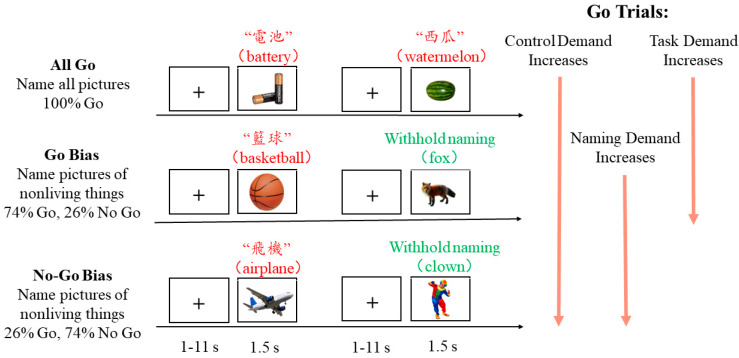
Task design. Overview of the categorical semantic Go/No-Go picture naming task. Examples of Go and No-Go trials for each of the three conditions: All Go, Go Bias, and No-Go Bias. Correct names for the two No-Go trials (withhold naming, in green) are fox and clown, respectively. Control demand for Go trials increases from the All Go to Go Bias to No-Go Bias condition. Task demand increases from All Go to Bias conditions. Naming demand increases from Go Bias to No-Go Bias conditions.

A semantic Go/No-Go picture naming task was performed by all participants in the MRI scanner using their native language, Cantonese. Photographs were displayed to participants, who were instructed to promptly and accurately name the photograph. Control demand during production was manipulated by varying the proportion of Go trials (i.e., naming trials) and No-Go trials (i.e., trials that inhibited responses were needed) across three conditions: All Go, Go Bias, and No-Go Bias (see Figure 1 for the task illustration). Specifically, in the All Go condition, participants were required to name all photographs (100% Go trials), and this was always administered first to minimize the possible influence of subsequent Go/No-Go task demands. In the Go Bias and No-Go Bias conditions, participants were instructed to name the photograph only if the photograph indicates a nonliving object (e.g., basketball) but inhibit their response if the photograph indicates a living object (e.g., fox). In the Go Bias condition, 74% of trials were Go trials, while 26% were No-Go trials. In the No-Go Bias condition, 26% of trials were Go trials, while 74% were No-Go trials. Control demands involved in production increased as the proportion of Go trials decreased from All Go to Go Bias and No-Go Bias. Furthermore, since the Go/No-Go decision was unnecessary in the All Go condition, the specific task demand escalated from the All Go condition to the Bias condition. In the No-Go Bias condition that contains a majority of No-Go trials (i.e., 74%), a prepotent inhibition tendency needs to be overcome when responding to Go trials. Therefore, though the task demands within two Bias conditions are consistent, the naming demand increased from the Go Bias condition to the No-Go Bias condition as the number of Go trials decreased. Prior to the formal scanning, a practice run similar to the real task but with different photographs was conducted in a simulator to ensure participants’ adaptation to the environment and the experimental task. In the scanner, participants always performed the All Go runs first before being informed regarding the Go/No-Go conditions.

Photographs were selected from two picture databases [84,85] and open-access online resources, including a broad range of common objects (e.g., people, animals, food, tools, nature, music instruments, clothes, transportations, and household items). We recruited 17 healthy, native Cantonese speakers who were not involved in the MRI experiment to provide Cantonese names for the selected photos. Only items with naming consistency higher than 65% were included in the final stimulus set with 318 colored photographs in total. All the selected stimuli were assigned to three conditions, with 106 unique items per condition (All Go, 78 nonliving and 28 living; Go Bias, 78 nonliving and 28 living; and No-Go Bias, 28 nonliving and 78 living). Target word frequency was obtained from the Cantonese-based Sketch Engine database [86]. Living and nonliving stimuli across three conditions were comparable in word length, word frequency, and name agreement (*p*s > 0.2).

Colored stimuli photographs with white backgrounds, lasting 1.5 s each, were displayed on a monitor with a variable inter-stimulus interval (ISI) ranging from 1 to 11 s (mean ISI = 4.08 s) to optimize the hemodynamic response using the *Optseq2* method [87]. For each photograph, participants were instructed to speak out its name or withhold their response based on the requirements for each condition. The responses were encouraged to be a single word without any additional comments. In the MRI scanner, participants completed a total of 6 runs, with 2 runs in each condition. During the scan, overt verbal responses were recorded and filtered using a dual-channel, MR-compatible, fiber optic microphone system provided by Optoacoustics Ltd. in Or-Yehuda, Israel. After the scan, participants were asked to rename all the photographs displayed earlier in a silent room to confirm their knowledge of the object names in situations where they refrained from responding (i.e., during No-Go trials).

### 2.3. Acquisition of MRI Data

We used a 3T Siemens Prisma MRI scanner with a 32-channel head coil to collect MRI data. T1-weighted images with anterior and posterior commissures identified were collected using a magnetization-prepared rapid acquisition gradient echo (MP-RAGE) sequence as anatomical references (Repetition Time [TR] = 2300 ms; Echo Time [TE] = 2.28 ms; Inversion Time [TI] = 900 ms; flip angle = 8°; echo spacing = 7 ms; acceleration factor = 2; field of view [FOV] = 256 mm^2^; voxel size = 1 × 1 × 1 mm; 160 contiguous slices).

Functional images relying on the BOLD response were collected using an echo-planar imaging (EPI) sequence (TR = 2500 ms; TE = 25 ms; flip angle = 90°; echo spacing = 0.49 ms; FOV = 240 mm^2^; voxel size = 3 × 3 × 3 mm; 41 contiguous axial slices, parallel to the AC–PC line, interleaved acquisition). Two additional volumes obtained as the first two volumes of each run were deleted for steady-state equilibrium, resulting in 128 volumes (320 s) in total for each of the six function runs.

### 2.4. Behavioral Data Analyses

Language entropy is a newly developed method for assessing the diversity of language usage in multilinguals [52,53,62,63]. The calculation of language entropy was based on Shannon entropy, which is a classical measure of information diversity and uncertainty [88]. In the current study, language entropy was calculated based on three questions from the LHQ. These questions assessed language use experience in 17 different communicative contexts via the time spent in each language (including social media, reading, writing, and speaking with different people) and the frequency of language activities on a 7-point scale (including different types of self-engaged activities). Language entropy in each context was first calculated with the following equation: $H=-\sum_{i=1}^{n}P_{i}{log}_{2}\left(P_{i}\right)$ using the *LanguageEntropy* package [89] in the R environment [90]. In this formula, *n* represents the total possible languages within a context, and *P_i_* is the proportion that language*_i_* is used within the context. The mean entropy was then calculated across all contexts for each participant. Lower language entropy values indicate an unbalanced and less diverse use of different languages, whereas higher entropy values indicate a more balanced and mixed use of each language, leading to higher uncertainty for which language will be used.

For the in-scanner picture naming task, responses were coded based on the recordings from the scan. Since we were mainly interested in language production, behavioral and fMRI analyses were only conducted on the Go trials, which represent word retrieval and production processes. To give readers some information regarding the No-Go trials, we added Supplementary Figure S1 (on the osf site) to demonstrate the commission error rate (failure to inhibit responses) difference between the two Bias conditions. In all conditions, responses were coded as correct if they were consistent with the target names (e.g., lemon for lemon) or acceptable alternatives that matched the photographs and were from the same category as the target word (e.g., lime for lemon). Responses that did not match the photographs (e.g., ice for tea) or trials without response were marked as incorrect.

It is essential to note that previous studies have demonstrated significant differences in behavioral performance and neural responses for naming living and nonliving things [91,92,93,94]. Therefore, to avoid any potential confounding effects of different categories (i.e., living vs. nonliving) and to minimize the discrepancy in numbers of Go trials, between the All Go and Bias conditions, we focused exclusively on the Go trials of nonliving things in our study (i.e., a subset of Go trials in the All Go condition, and all Go trials in the Bias conditions). This action only affected the number of trials in the All Go condition included in the analysis. Yet, the analysis and results, including all trials in the All Go condition, are comparable with the subset analysis (see Supplementary Figure S2 on the osf site).

The incorrect response rate of the Go trials was calculated for each condition by dividing the number of incorrect responses by the total number of nonliving Go trials in the corresponding condition. To effectively demonstrate how production performance changed as control demand increased, the categorical conditions for control demand were systematically coded on a numeric scale (i.e., All Go is 1, Go Bias is 2, and No-Go Bias is 3). Since the incorrect response rates were calculated based on each task condition, a generalized linear model was conducted on the incorrect response rate to analyze the effects of control demand and language diversity and their interaction, employing the *glm* function with a Gaussian family from the *stats* package in the R environment [95].

To calculate the reaction times (RTs) for the Go trials, the response onsets of the recordings provided in the scanner were extracted using customized *Praat* scripts [96], which could detect response onsets based on the pitch deviations of the filtered auditory signal. All these onsets were double-checked based on both the audio and visual speech streams. The RTs of Go trials were calculated by subtracting the photograph onsets (from E-Prime output) from the response onsets. Incorrect trials and those whose reaction times exceeded 2.5 standard deviations were excluded. Categorical task conditions were also coded on a numeric scale. To investigate the main effects of task conditions and language entropy and their interaction, a mixed-effects regression was conducted on trial-level RTs employing the *lmer* function in the *lme4* package [97]. We also included random intercepts and slopes attributable to different participants, as well as random intercepts related to the target words in the model, as suggested by Barr and Levy [98]. The *p* values for regression coefficients were obtained using the *lmerTest* package [99].

### 2.5. fMRI Data Analyses

Prior to data processing, the data quality was assessed using the fBIRN QA tool [100], measuring the mean signal fluctuation to noise ratio (SFNR), the number of potentially clipped voxels, and per-slice variation. Additionally, the functional and anatomical images were visually inspected for artifacts and signal drop-out. Brain structure was extracted using the Optimized Brain Extraction for Pathological Brains script (optiBET) [101], with the skull and other non-brain tissues separated and stripped out. We used FSL (Version 6.0.5) with FEAT (fMRI expert analysis tool) Version 6.0 [102,103] for further processing and analyses. Procedures, including motion correction (FSL MCFLIRT), B0 unwarping with field mapping, slice timing correction, spatial smoothing (with FWHM equal to 5 mm) high-pass filtering, coregistration, and normalization, were also conducted. When modeling the BOLD signal for each event, we employed a double-gamma hemodynamic response function, and only correct trials were included in the analyses. The first-level analyses were conducted on each participant’s individual runs, in which standard motion parameters were included as confounding EVs (Explanatory Variables) to eliminate any motion effects that may persist after motion correction. Then group-level analyses using FMRIB’s local analysis of mixed effects were conducted across runs and participants (FLAME 1+2) [103,104]. Specifically, we identified regions responsive to nonliving Go trials across different conditions, respectively (i.e., Go trials in All Go, Go Bias, and No-Go Bias conditions), compared with the implicit baseline of each participant. To investigate the effect of control demands, we then compared parametric functional activation across task conditions for Go trials (All Go < Go Bias, All Go < No-Go Bias, Go Bias < No-Go Bias). Conditional differences were all masked by the condition with higher control demands. Furthermore, to assess the main effect of language entropy, we collapsed nonliving Go trials from all conditions for each participant and correlated their activation with the corresponding language entropy deviation of each participant. The activation maps for the language entropy correlation were masked by the contrast reflecting overall Go trial activation. Significant activations were identified through a two-step process. Initially, Z-statistical images (Gaussianized T/F) were voxel-level thresholded at *p* < 0.01. To account for multiple comparisons, clusters of voxels were corrected following Gaussian random field theory [105] at a corrected threshold of *p* = 0.05. The estimated significance level of each cluster was compared with the cluster probability threshold, and only clusters surpassing this threshold were included in the results [106].

Additional analyses were conducted to estimate how language entropy modulated the effect of control demand (i.e., the interaction between language entropy and control demand). Specific to Go trials, we correlated participants’ language entropy with changes in brain activation from the All Go condition to the Go Bias condition (Go Bias > All Go), the All Go condition to the No-Go Bias condition (No-Go Bias > All Go), and the Go Bias to the No-Go Bias condition (No-Go Bias > Go Bias). Positive correlations (i.e., higher language entropy associated with larger increases in activation) would indicate that participants with greater language diversity tend to be more affected by the increased control demand. On the other hand, negative correlations (i.e., higher language entropy associated with smaller increases in activation) would suggest that participants with greater language diversity are less affected by the increased control demand. These negative correlations would then reflect that multilingual experience can help multilinguals better cope with increased control demands. To ensure that only meaningful differences were included in the analyses, the interaction between language entropy and control demand was masked with activation triggered by changes in control demand (e.g., the effect of language entropy on changes in brain activation from the All Go to the Go Bias condition was masked with the activation map comparing the All Go with the Go Bias condition). All results were overlayed on a representative brain in MNI space and reported using MNI coordinates. The Harvard-Oxford Structural Atlas [107] was used to identify the regions of the whole brain.

### 2.6. Functional Connectivity Analyses

To investigate the network connectivity status modulated by language entropy at different task demands, we analyzed task-based language network segregation. We started by creating 264 sphere nodes (5 mm radius) across the whole brain based on the coordinates identified by Power and Cohen [108]. Then we divided these ROIs into 14 networks, including language-specific networks (Left language network and Right language homologous network) and networks related to other general functions (Cingulo-opercular/Fronto-parietal control, Ventral/Dorsal attention, Hand/Mouth somatomotor, Visual, Salience, Auditory, Subcortical, Cerebellar, and Default), using similar methods as in the previous study [109]. The language networks selected were based on Fedorenko and Hsieh [110], and other networks were from Power and Cohen [108]. Thirty-three nodes that cannot fit with any network were excluded from the analysis. The final set was compromised with 231 nodes across 14 networks (see Supplementary Table S1 on the osf site).

Functional connectivity data were analyzed using the CONN functional connectivity toolbox (Version 18.a) under MATLAB environment [111]. For each condition of each participant, the fully processed time-series data of each node was extracted and correlated with the time-series of every other node. A Fisher-Z transformation was conducted on all correlations. Negative correlations were excluded from further analysis due to the uncertainty of their meaning. Within-network connectivity was obtained by averaging the node-to-node correlation of each node pair in the target network (i.e., diagonal blocks). Between-network connectivity was the average correlation between each node in a network and all other nodes outside of that network (i.e., off-diagonal blocks). Finally, left-language network segregation was calculated as the difference between the within-network connectivity and between-network connectivity of the language network divided by the within-network connectivity [112,113].

Consistent with the analysis for fMRI activation, we also investigated the effects of task demand and language entropy, as well as their interaction, on language network segregation separately. It should be noted that since network connectivity reflects the brain states throughout the whole run and the Go Bias and No-Go Bias conditions reflect similar task demands, we combined the two Bias conditions in the analyses. Mixed-effect regressions were conducted using the *lmer* function in the *lme4* package [97] in the R environment [95].

## 3. Results

### 3.1. Behavioral Results

#### 3.1.1. Error Rates

**Figure 2:**
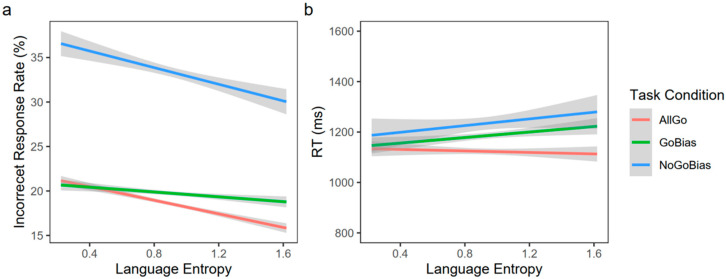
Behavioral results for the nonliving Go trials in Go/No-Go picture naming task. (**a**) Incorrect response rates for nonliving Go trials across conditions. Higher language entropy was associated with lower incorrect response rates. Incorrect response rates increased as control demand increased. (**b**) Reaction times (RTs) for nonliving Go trials across conditions. RTs were significantly longer as control demand increased. Yet, the main effect of language entropy and its interaction with control demand were not significant.

To investigate the effect of control demand and language entropy on language production, a generalized linear regression was conducted on the incorrect response rates of nonliving Go trials for each participant across the three conditions (All Go, Go Bias, and No-Go Bias; Table 2 and Figure 2a). Results showed a main effect of control demand (*β* = 5.99, SE = 0.12, *p* < 0.001), manifesting significantly higher incorrect response rates as task difficulty increased (i.e., All Go < Go Bias < No Go Bias; All Go vs. Go Bias: *β* = −1.25, SE = 0.17, *p* < 0.001; All Go vs. No-Go Bias: *β* = −14.81, SE = 0.24, *p* < 0.001; Go Bias vs. No-Go Bias: *β* = −13.57, SE = 0.24, *p* < 0.001). There was also a significant main effect of language entropy, such that the incorrect response rates for participants with lower language entropy were significantly higher than those with higher language entropy (*β* = −0.96, SE = 0.23, *p* < 0.001). Yet, there was no significant interaction between language entropy and control demand (*β* = 0.07, SE = 0.12, *p* = 0.53). Therefore, results from incorrect response rates suggested that higher language entropy was beneficial for native language production across different control demands.

#### 3.1.2. Reaction Times (RTs)

A linear mixed-effect model was conducted on RTs of nonliving Go trials to explore the effect of control demand and language entropy on the speed of language production (see Table 2 and Figure 2b). Results revealed a significant main effect of control demand (*β* = 56.77, SE = 17.57, *p* < 0.01), such that RTs were different among the three task conditions (i.e., All Go < Go Bias < No-Go Bias; All Go vs. Go Bias: *β* = −50.58, SE = 26.17, *p* = 0.05; All Go vs. No-Go Bias: *β* = −116.28, SE = 36.77, *p* < 0.01; Go Bias vs. No-Go Bias: *β* = −65.70, SE = 35.04, *p* = 0.06). There was no significant main effect of language entropy on RTs (*β* = −9.14, SE = 24.84, *p* = 0.72). Also, the interaction between language entropy and condition was not significant (*β* = 11.05, SE = 9.38, *p* = 0.25). These results suggested that language production performance declined as the task became more challenging, although language entropy did not have a significant effect on reaction times.

### 3.2. fMRI Activation Results

#### 3.2.1. Basic Pattern of Activation

The Go trials in all conditions (i.e., All Go, Go Bias, and No-Go Bias) elicited similar activation patterns in regions that were known to be involved in language and cognitive processing, including bilateral frontal pole, superior, middle, and inferior frontal gyri, frontal orbital cortex, pre- and post-central gyri, superior, middle, and inferior temporal gyri, angular gyri, precuneus cortex, supramarginal gyri, lateral occipital cortex, occipital pole, as well as bilateral cingulate cortex and insular gyri (see Supplementary Figure S3 and Supplementary Table S2 on the osf site).

#### 3.2.2. Main Effects of Control Demand

**Figure 3:**
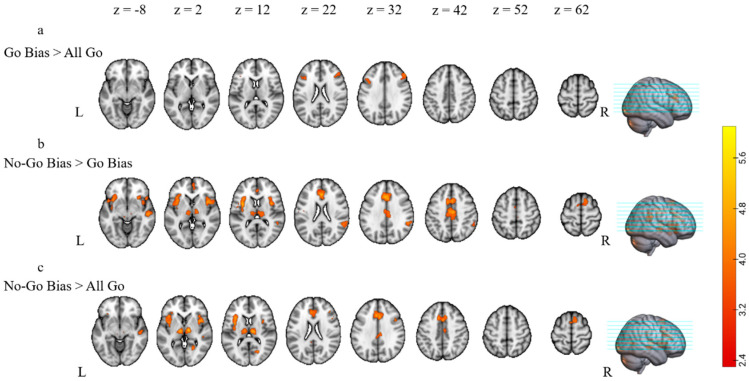
fMRI activation as a function of control demand. Overview of the regions in which there were significant increases in activation of (**a**) Go Bias condition > All Go condition, (**b**) No-Go Bias condition > Go Bias condition, and (**c**) No-Go Bias condition > All Go condition. Slices are depicted in increments of 10 mm, starting at z = −8 and ending at z = 62. Activations were reported for clusters that had a corrected *p*-value of *p* < 0.05 at the cluster level.

To reveal the neural response sensitive to increased control demand, we compared the functional activation of nonliving Go trials across three conditions as a function of increased demands (i.e., All Go < Go Bias, Go Bias < No-Go Bias, All Go < No-Go Bias). Participants showed greater activation during the Go Bias condition compared with the All Go condition in the right frontal pole, extending to the bilateral middle and inferior frontal gyri and the left precentral gyri (Table 3 and Figure 3a). The No-Go Bias condition elicited greater activation compared with the Go Bias in bilateral orbitofrontal cortex, bilateral operculum cortex, bilateral inferior frontal gyri, right hemisphere regions including superior frontal gyrus, precentral gyrus, superior and middle temporal gyri, supramarginal gyrus, and angular gyrus, as well as bilateral cingulate cortex and insular cortex (Table 3 and Figure 3b). Similar activation patterns were also found when comparing the Go trials in the No-Go Bias condition and the All Go condition. Compared with the All Go condition, the No-Go Bias condition showed increased activation in bilateral orbitofrontal and operculum cortex, bilateral inferior frontal gyri extending to the right superior and middle frontal gyrus, right superior and middle temporal gyri, right precuneus cortex, right lingual gyrus, as well as bilateral cingulate cortex and insular cortex (Table 3 and Figure 3c). In general, as control demand increased, participants elicited greater activation in regions related to language processing as well as domain-general control.

#### 3.2.3. Main Effect of Language Entropy

**Figure 4:**
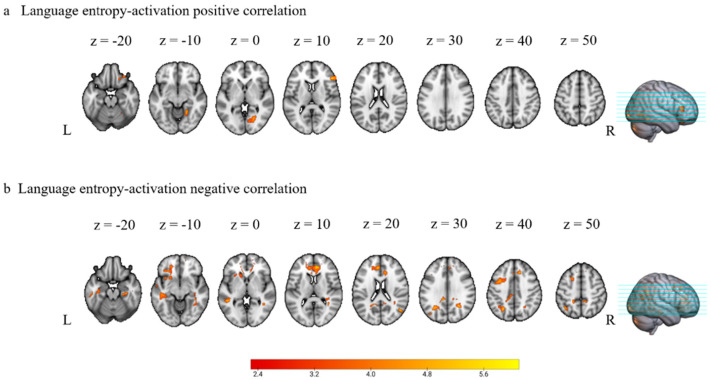
Correlations between language entropy and fMRI activation during production. (**a**) Regions in which positive correlations were found, i.e., regions in which higher language entropy values were associated with more activation. (**b**) Regions in which negative correlations were found, i.e., regions in which higher language entropy was associated with less activation. Activations were reported for clusters that had a corrected *p*-value of *p* < 0.05 at the cluster level.

To investigate the effect of language entropy, we correlated the functional activation across all nonliving Go trials for each participant with their demeaned language entropy score. Positive correlations were found in the right hemisphere, including the inferior frontal gyrus, orbitofrontal cortex, lingual gyrus, and occipital cortex (Table 4 and Figure 4a). Negative correlations were found between language entropy and brain activation in the left superior and middle frontal gyri, bilateral anterior cingulate and paracingulate gyri, left precentral gyri and right postcentral gyri, left superior parietal lobule, left superior, middle, and inferior temporal gyri, left supramarginal gyrus, bilateral angular gyri, and bilateral occipital cortex (Table 4 and Figure 4b).

#### 3.2.4. Interaction between Language Entropy and Control Demand

**Figure 5:**
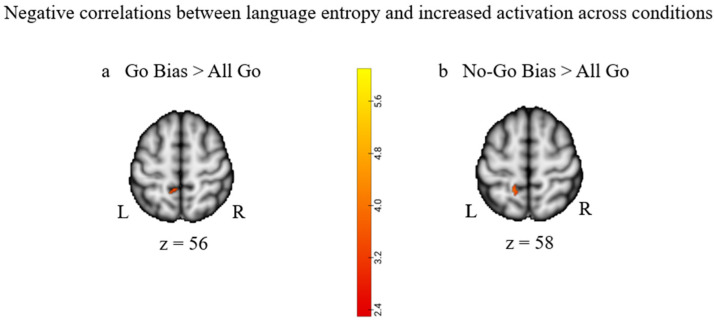
Negative correlations between language entropy and increased activation across conditions: (**a**) Negative correlation between language entropy and increases in activation between the All Go and Go Bias conditions (All Go < Go Bias). (**b**) Negative correlation between language entropy and increases in activation between the All Go and No-Go Bias conditions (All Go < No-Go Bias). Activations were reported for clusters that had a corrected *p*-value of *p* < 0.05 at the cluster level.

To investigate how language diversity modulated the functional response to increased control demand (i.e., language entropy × control demand interaction), we correlated participants’ language entropy with changes in brain activation as a function of control demand (i.e., All Go < Go Bias < No-Go Bias). Higher language entropy was found to be associated with smaller increases (i.e., negative correlation) in activation from the All Go to the Bias conditions in the left precuneus cortex and left pre- and postcentral gyri (see Table 5 and Figure 5). No significant relationship with language entropy was found when comparing the Go Bias and No-Go Bias conditions.

### 3.3. Functional Connectivity Results

#### 3.3.1. Main Effect of Task Demand

**Figure 6:**
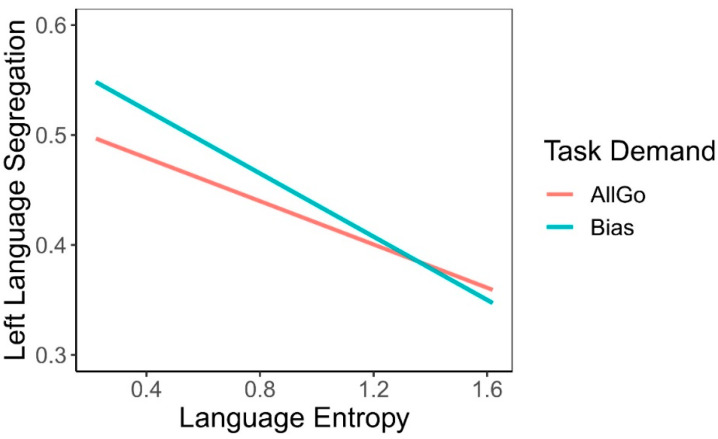
The relationship between language segregation, language entropy, and task demand. Higher language entropy was associated with lower language network segregation. Yet, the main effect of task demand and its interaction with language entropy were not significant.

Mixed-effect regression with random intercepts and slopes for different participants showed that there was no significant main effect of task demand on language network segregation (Figure 6, *β* = 0.02, SE = 0.01, *p* = 0.14). This result indicated that language network segregation during language production was relatively consistent despite the differences in task demand.

#### 3.3.2. Main Effect of Language Entropy

Regression analyses on language network segregation showed a significant main effect of language entropy (Figure 6, *β* = −0.04, SE = 0.013, *p* < 0.01), such that higher language entropy was associated with lower language network segregation. This relationship suggested that individuals with more diverse language use experience showed a broader involvement of functional networks during native language production.

#### 3.3.3. Interaction between Language Entropy and Task Demand

Regression results showed that there was no significant interaction between language entropy and task demand on language network segregation (Figure 6, β = −0.02, SE = 0.01, *p* = 0.09). This, combined with the main effect of entropy, suggested that individuals’ multilingual experience independently influenced functional connectivity patterns during tasks which were not necessarily modulated by task demands.

## 4. Discussion

In the present study, we investigated how the experience of using multiple languages affected native language production in the context of manipulating control demands, proving evidence from behavioral aspects as well as fMRI activation and functional connectivity. There are a few highlights in the current study. First, we treated multilingual language experience as a continuous variable in a dynamic and integrated way rather than using the conventional categorical classification. Second, while the majority of previous studies focused on bilinguals, we tested multilinguals who spoke at least three languages, further supplementing and enhancing the previous literature. We found that a diverse and balanced multilingual experience enhanced multilinguals’ native language production and was associated with greater involvement of other networks. Additionally, multilinguals with higher language entropy were better able to cope with increased task demands in language production. That is, our results suggest that the experience of using multiple languages extensively is beneficial for maintaining the native language. Below, we discuss these results in detail.

First, increased control demand significantly affected native language production both behaviorally and neurally. Specifically, as control demand increased (All Go < Go Bias < No-Go Bias), participants showed increased incorrect response rates, longer reaction times, and increased brain activation in bilateral cognitive control and language-related regions (see Table 3 and Figure 3 for more details). These results are consistent with previous studies that manipulated control demands in language production [13,66,68,69,114,115]. In response to increased control demands, multilinguals recruited more neural resources from the bilateral inferior and middle frontal gyri, the left superior and middle temporal gyri, and the bilateral insular cortex. These regions are part of the well-established left frontal-parietal-temporal language-related circuits and homologous regions in the right hemisphere [116,117,118,119,120,121,122,123]. Additionally, regions related to domain-general cognition, such as the bilateral cingulate and prefrontal cortex, were more activated in response to the increased control demand in the current study [124,125,126,127,128,129,130,131]. All these results suggest that multilinguals’ native language production under higher control demands involves additional domain-specific and domain-general neural resources.

Furthermore, the main focus of the current paper is to investigate the effect of multiple language use experience measured by language entropy. Behaviorally, higher language entropy was associated with better L1 production, reflected by lower incorrect response rates. Yet, there was no significant effect of language entropy on reaction times, which is likely due to a speed-accuracy trade-off where multilinguals with higher language entropy tended to sacrifice naming speed for response accuracy. Neurally, individuals with higher language entropy showed less activation across many regions that are typically involved in language processing (e.g., left temporal cortex, left precentral and right postcentral gyri, bilateral anterior cingulate cortex, bilateral angular gyri). Along with the enhanced behavioral performance in multilinguals with higher language entropy, these results suggest that the diverse and balanced use of multiple languages benefits multilinguals’ native language production and its neural efficiency (i.e., using fewer neural resources to achieve enhanced behavioral performance). The current findings offer a new perspective on the traditional view regarding L1 attrition and suggest that the use of multiple languages could be beneficial for the maintenance of one’s native language. Yet, unlike bilinguals who lost contact with their native language, multilinguals in the present study still maintained continuous exposure to their native language. This raises the possibility that multilingual experience may have different implications for native language production across different multilingual groups. Moreover, the differences between the observed facilitation and L1 attrition further demonstrate that exposure to both the native language and other languages is important for the maintenance of native language ability [42,43,132].

In addition to the negative correlations between language entropy and brain activation, higher language entropy was also associated with greater activation in regions related to cognitive control (e.g., the right inferior frontal gyrus). Consistently, functional connectivity results showed that individuals with higher language entropy had lower language network segregation, indicating greater involvement of other brain networks during language production. These entropy-brain relationships might reflect a strategy shift such that multilinguals with more extensive use of multiple languages employ more domain-general (i.e., more activation in frontal regions) and less domain-specific (i.e., less activation in language regions) neural resources during native language production.

Furthermore, individuals with varying degrees of multilingualism exhibited differences in their ability to regulate task demands during their native language production. While there was no statistically significant interaction observed on behavioral performance, multilinguals with higher language entropy were less affected by increased control demand during production, as evidenced by their neural efforts. Specifically, when comparing Bias conditions with the All Go condition, higher language entropy was steadily associated with smaller increases in activation in the left postcentral gyrus. As the site where the primary sensory cortex is located, the left postcentral gyri has been found to be related to successful overt naming [69,70]. Moreover, it has also been shown to be critical in modulating attentional processing demands [133,134]. It is worthwhile to note that the modulation effect of language entropy on the effect of control demand during production was only present when comparing the Bias conditions with the All Go condition. As suggested in previous studies, the switch from the All Go to the Bias conditions may reflect increased task demands (i.e., additional Go/No-Go tasks in the Bias conditions), whereas the difference between the Go and No-Go Bias conditions might reflect a change in the domain of naming demands (i.e., more cognitive resources were taken to overcome the prepotent inhibition tendency and name the target photos in the No-Go Bias condition) [69]. Therefore, the results suggest that the modulation effect of multiple language use experiences might be strongest when there is an increase in general task demands but not when there is an increase in naming demands. Recall that, in general, higher language entropy was associated with less activation in language-related brain regions. Together with the interaction effects, all these results suggest that the multiple language experience is beneficial for native language production in general, especially in the context of higher task demands.

Although our results shed light on the influence of control demands, multilingual experience, and their interaction on native language production, there are a few limitations. First, though we used language entropy to quantify the usage of multiple languages in a more comprehensive and context-specific way, the calculation of language entropy currently relies on subjective reports. Future research could seek more objective measures to reflect multilinguals’ language experience. Second, the gender distribution of participants was unbalanced. Though previous studies have suggested that gender plays a trivial role in brain function, cognition, and behavior (d = 0.20 or d ≤ 0.10) [135,136], future studies should target a more balanced sample of different genders when possible.

## 5. Conclusions

In conclusion, our findings demonstrate that multilinguals are sensitive to the increased control demands during native language production, showing poorer behavioral performance and more extensive brain activation. Importantly, a more balanced and diverse language use experience facilitated native language production both behaviorally and neurally. With more extensive use of multiple languages, multilinguals might undergo a strategy shift such that they tend to employ more domain-general and less domain-specific neural resources during native language production. Additionally, the diverse and balanced use of multiple languages was beneficial for multilinguals in dealing with increased task demands in native language production.

## Figures and Tables

**Table 1:** Participants’ demographic characteristics and neuropsychological testing scores.

	Mean	SD
Demographic Characteristics		
N	36	
Age (years)	21.1	1.8
Education (years)	15.1	1.7
Gender (F/M)	27/9	
Neuropsychological testing ^1^		
MoCA (Score out of 30)	28.78	1.16
GDS-15	2.44	1.98
Color Vision (Score out of 25)	24.03	1.42
Forward Digit Span (Score out of 8)	7.31	0.74
Backward Digit Span (Score out of 7)	5.00	1.41
Vocabulary (WAIS-RC) (Score out of 40)	27.78	5.87
Reading Habits (Score out of 35)	20.61	4.17
VF_Cantonese (Animal + Vegetable, Correct token)	31.72	5.88
VF_Mandarin (Fruit, Correct token)	13.14	3.25
VF_English (Occupation, Correct token)	8.81	3.53
Simple Speed (ms)	259.41	38.56
Complex Speed (ms)	292.86	60.05
AX-CPT: AY RT (ms)	378.70	51.70
AX-CPT: AY ER (out of 1)	0.19	0.17
Stroop Effect (ms) (Incongruent-Congruent)	22.68	70.82

^1^ Neuropsychological testing included a Montreal Cognitive Assessment (MoCA) to assess general cognitive states [72,73]; a 15 items geriatric depression scale (GDS-15, a shortened version of the Geriatric Depression Scale) which has been verified as a reliable measure of depression in younger and older populations [74,75]; a Color Vision test; Wechsler Adult Intelligence Scale (WAIS) digit span tasks with forward and reverse sets to assess working memory [76]; a vocabulary test from the WAIS revised by China (WAIS-RC) to gauge vocabulary knowledge [77]; a reading habits questionnaire [78]; a categorical verbal fluency task (VF) using different semantic categories as another assessment of vocabulary in each language [79]; a simple and choice processing speed task to assess speed; an AX continuous performance task (AX-CPT) [80,81], and a Stroop task to assess executive functions [82,83]. All these tasks were administered and performed in Mandarin Chinese. SD represents standard deviation.

**Table 2:** Behavioral results on nonliving Go trials (incorrect response rate and reaction times).

	Incorrect Response Rate (%)	Reaction Times (ms)
All Go	18.5 (5.7)	1127.57 (279.77)
Go Bias	19.8 (6.5)	1182.52 (288.13)
No-Go Bias	33.3 (9.0)	1229.99 (313.96)

The incorrect response rate of the Go trials was calculated for each condition by dividing the number of incorrect responses by the total number of nonliving Go trials in the corresponding condition. Values provided are means, with standard deviations in the parentheses.

**Table 3:** fMRI activation as a function of control demand.

			Hemisphere	Voxels	Coordinates (mm)			Z Value
					x	y	z	
**Go Bias > All Go**								
	**Frontal pole**		**Right**	**327**	**46**	**36**	**32**	**3.27**
		Middle frontal gyrus	Right		50	32	32	4.06
		Inferior frontal gyrus	Right		50	22	30	3.43
	**Middle frontal gyrus**		**Left**	**250**	**−46**	**16**	**34**	**4.19**
		Inferior frontal gyrus	Left		−42	12	34	4.00
		Precentral gyrus	Left		−40	10	32	3.37
**NoGo Bias > Go Bias**								
	**Frontal orbital cortex**		**Left**	**1477**	**−34**	**18**	**−8**	**3.47**
		Frontal operculum cortex	Left		−34	20	10	4.36
		Inferior frontal gyrus	Left		−40	18	10	3.15
		Central opercular cortex	Left		−36	6	12	4.97
		Insular cortex	Left		−34	8	8	4.25
	**Frontal orbital cortex**		**Right**	**1081**	**30**	**22**	**−8**	**4.12**
		Frontal operculum cortex	Right		38	20	6	4.17
		Inferior frontal gyrus	Right		54	14	6	4.07
		Central opercular cortex	Right		38	6	10	4.58
		Precentral gyrus	Right		52	6	6	3.59
		Insular cortex	Right		36	6	8	4.79
	**Anterior cingulate gyrus**		**Left**	**2660**	**−2**	**20**	**34**	**5.04**
		Anterior cingulate gyrus	Right		4	16	36	5.45
		Paracingulate gyrus	Left		−10	18	42	3.60
		Paracingulate gyrus	Right		8	18	42	4.01
	**Superior frontal gyrus**		**Right**	**309**	**10**	**16**	**60**	**4.57**
	**Superior temporal gyrus**		**Right**	**265**	**52**	**−14**	**−8**	**3.54**
		Middle temporal gyrus	Right		56	−18	−6	4.69
	**Angular gyrus**		**Right**	**539**	**60**	**−46**	**24**	**4.55**
		Supramarginal gyrus	Right		64	−44	24	3.48
**NoGo Bias > All Go**								
	**Frontal orbital cortex**		**Left**	**894**	**−38**	**22**	**2**	**4.48**
		Inferior frontal gyrus	Left		−46	18	2	3.48
		Frontal operculum cortex	Left		−34	16	10	5.09
		Central opercular cortex	Left		−44	8	2	3.75
		Insular cortex	Left		−34	4	10	4.86
	**Frontal orbital cortex**		**Right**	**352**	**38**	**24**	**2**	**3.49**
		Frontal operculum cortex	Right		44	12	2	4.60
		Central opercular cortex	Right		36	8	10	3.94
		Insular cortex	Right		38	10	4	4.88
	**Middle frontal gyrus**		**Right**	**172**	**52**	**24**	**28**	**3.94**
		Inferior frontal gyrus	Right		50	16	24	4.13
	**Anterior cingulate gyrus**		**Left**	**1089**	**−4**	**24**	**32**	**4.81**
		Anterior cingulate gyrus	Right		4	20	32	4.80
		Paracingulate gyrus	Left		−10	18	40	4.65
		Paracingulate gyrus	Right		4	26	30	4.69
	**Superior frontal gyrus**		**Right**	**238**	**12**	**4**	**66**	**4.53**
	**Superior temporal gyrus**		**Right**	**192**	**54**	**−14**	**−4**	**3.64**
		Middle temporal gyrus	Right		52	−26	−4	3.54
	**Posterior cingulate gyrus**		**Right**	**213**	**6**	**−30**	**26**	**4.28**
	**Precuneus cortex**		**Right**	**204**	**20**	**−54**	**4**	**3.31**
		Lingual gyrus	Right		20	−58	4	3.61

**Table 4:** Correlations between language entropy and fMRI activation during production.

			Hemisphere	Voxels	Coordinates (mm)			Z Value
					x	y	z	
**Positive correlation**								
	**Frontal orbital cortex**		**Right**	**68**	**22**	**32**	**−24**	**4.72**
	**Inferior frontal gyrus**		**Right**	**198**	**50**	**32**	**10**	**5.64**
	**Lingual gyrus**		**Right**	**451**	**22**	**−52**	**−12**	**5.08**
		Temporal occipital fusiform cortex	Right		26	−52	−16	4.83
		Occipital fusiform gyrus	Right		24	−68	−4	5.10
**Negative correlation**								
	**Anterior cingulate gyrus**		**Left**	**1795**	**−8**	**42**	**6**	**3.30**
		Anterior cingulate gyrus	Right		8	40	10	5.51
		Paracingulate gyrus	Left		−4	42	20	4.14
		Paracingulate gyrus	Right		4	36	10	4.20
	**Superior frontal gyrus**		**Left**	**42**	**−10**	**26**	**62**	**3.66**
	**Middle frontal gyrus**		**Left**	**408**	**−36**	**8**	**38**	**3.49**
		Precentral gyrus	Left		−46	0	38	5.05
	**Superior temporal gyrus**		**Left**	**41**	**−54**	**−2**	**−14**	**4.19**
	**Middle temporal gyrus**		**Left**	**398**	**−48**	**−28**	**−8**	**3.52**
		Temporal fusiform cortex	Left		−38	−30	−14	3.49
	**Temporal fusiform cortex**		**Right**	**131**	**30**	**−26**	**−22**	**3.19**
	**Inferior temporal gyrus**		**Left**	**71**	**−56**	**−32**	**−20**	**3.71**
	**Postcentral gyrus**		**Right**	**49**	**12**	**−40**	**52**	**3.11**
	**Posterior supramarginal gyrus**		**Left**	**808**	**−36**	**−54**	**58**	**3.48**
		Superior parietal lobe	Left		−36	−56	44	3.63
		Angular gyrus	Left		−40	−58	46	4.47
	**Angular gyrus**		**Right**	**97**	**46**	**−62**	**20**	**3.77**
		Lateral occipital cortex	Right		48	−64	20	4.87
	**Lateral occipital cortex**		**Left**	**56**	**−46**	**−70**	**26**	**4.36**

**Table 5:** Correlations between language entropy and increased activation across conditions.

			Hemisphere	Voxels	Coordinates (mm)			Z Value
					x	y	z	
**GoBias > AllGo: Negative correlation**								
	**Precuneus cortex**		**Left**	**15**	**−12**	**−48**	**56**	**3.50**
		Postcentral gyrus	Left		−12	−48	54	3.31
**NoGoBias > AllGo: Negative correlation**								
	**Postcentral gyrus**		**Left**	**29**	**−16**	**−46**	**58**	**3.80**
	**Occipital pole**		**Right**	**65**	**4**	**−90**	**14**	**3.49**

## Data Availability

Data are contained within the article and Supplementary Materials. The data presented in this study are openly available on OSF Platform at https://osf.io/wavnz/?view_only=e609e584222540b581b09b0316ade6f5 (accessed on 9 November 2023).

## Supplementary Materials

The following supporting information can be downloaded at: https://www.mdpi.com/article/10.3390/brainsci13111587/s1, Figure S1: Behavioral results for commission errors of No-Go trials; Figure S2: Behavioral results for all of the Go trials; Figure S3: Basic activation patterns across three conditions; Table S1: Network information for functional connectivity analyses; Table S2: Basic activation patterns across three conditions.
