# Supplementary material for: Multilingual Language Diversity Protects Native Language Production under Different Control Demands
Source: Brain Sci. 2023 Nov 13;13(11):1587. doi: 10.3390/brainsci13111587 (PMC10670415; doi:10.3390/brainsci13111587)
Supplement: Supplementary file 1 [file brainsci-13-01587-s001.zip › Figure S1 Behavioral Results for Commision Errors of No-Go Trials.pdf]

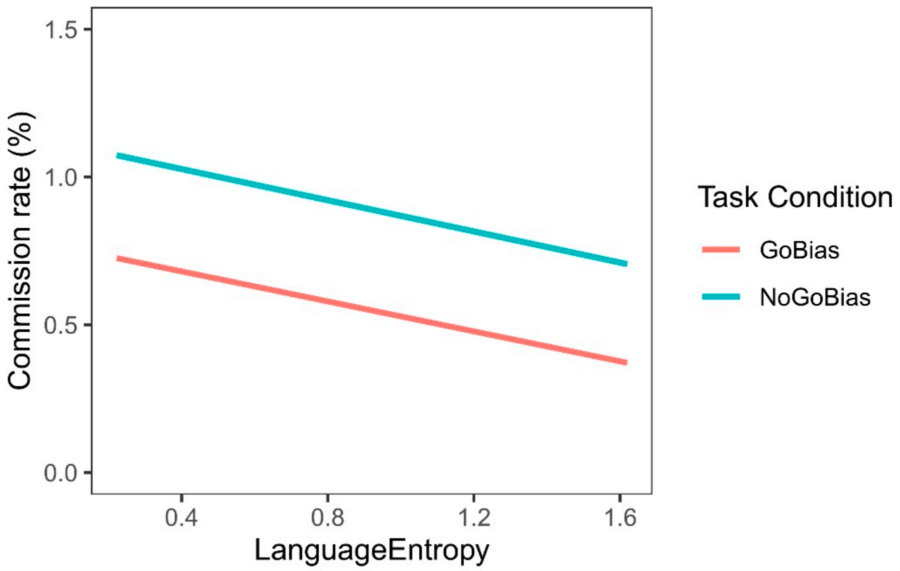

Supplementary Figure S1 *Behavioral results for the No-Go trials in Go/No-Go picture naming task. Commission error rates for No-Go trials across conditions. Commission errors refer to failures to inhibit a response on No-Go trial. The commission error rates were computed by dividing the commission errors by the total number of No-Go trials across conditions. Higher commission error rate in the No-Go Bias condition compared to the Go Bias condition. As language entropy increases, there showed a decrease trend of commission error rate.*
