# Supplementary material for: Multilingual Language Diversity Protects Native Language Production under Different Control Demands
Source: Brain Sci. 2023 Nov 13;13(11):1587. doi: 10.3390/brainsci13111587 (PMC10670415; doi:10.3390/brainsci13111587)
Supplement: Supplementary file 1 [file brainsci-13-01587-s001.zip › Figure S2 Behavioral Results for All of the Go Trials.pdf]

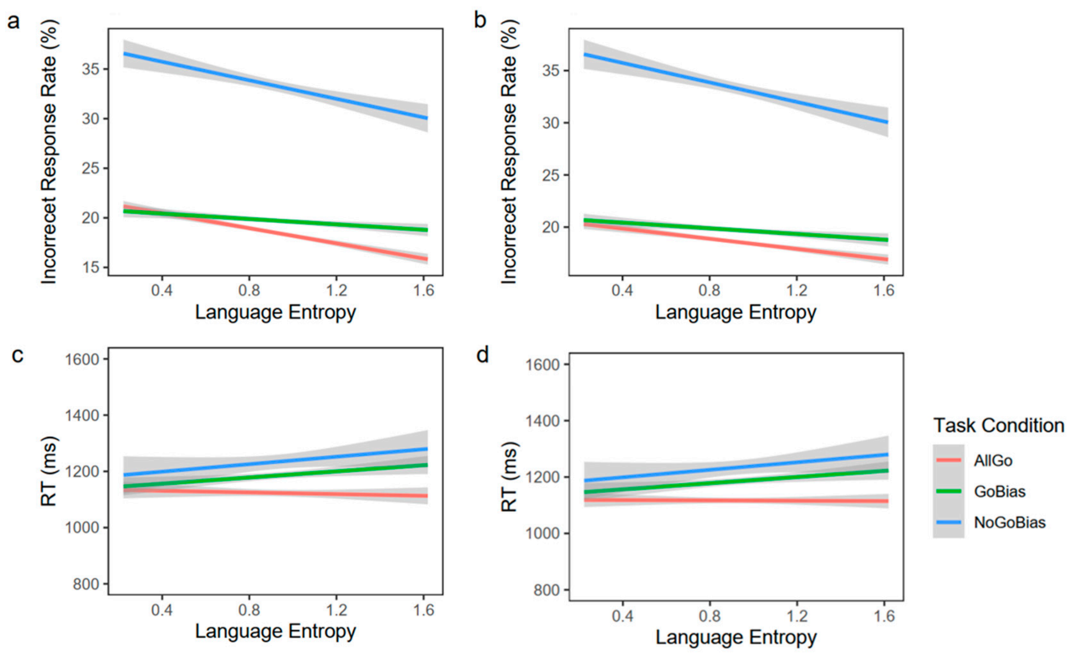

Supplementary Figure S2 *Behavioral results for the nonliving Go trials and the entire Go trials in Go/No-Go picture naming task. (a) Incorrect response rates for the nonliving Go trials across conditions. (b) Incorrect response rates for the entire Go trials across conditions. (c) Reaction times (RTs) for the nonliving Go trials across conditions. (d) Reaction times (RTs) for the entire Go trials across conditions. The behavioral results for nonliving Go trials showed similar patterns as the entire Go trials.*
