# Supplementary material for: Multilingual Language Diversity Protects Native Language Production under Different Control Demands
Source: Brain Sci. 2023 Nov 13;13(11):1587. doi: 10.3390/brainsci13111587 (PMC10670415; doi:10.3390/brainsci13111587)
Supplement: Supplementary file 1 [file brainsci-13-01587-s001.zip › Figure S3 Basic Activation Patterns across Three Conditions.pdf]

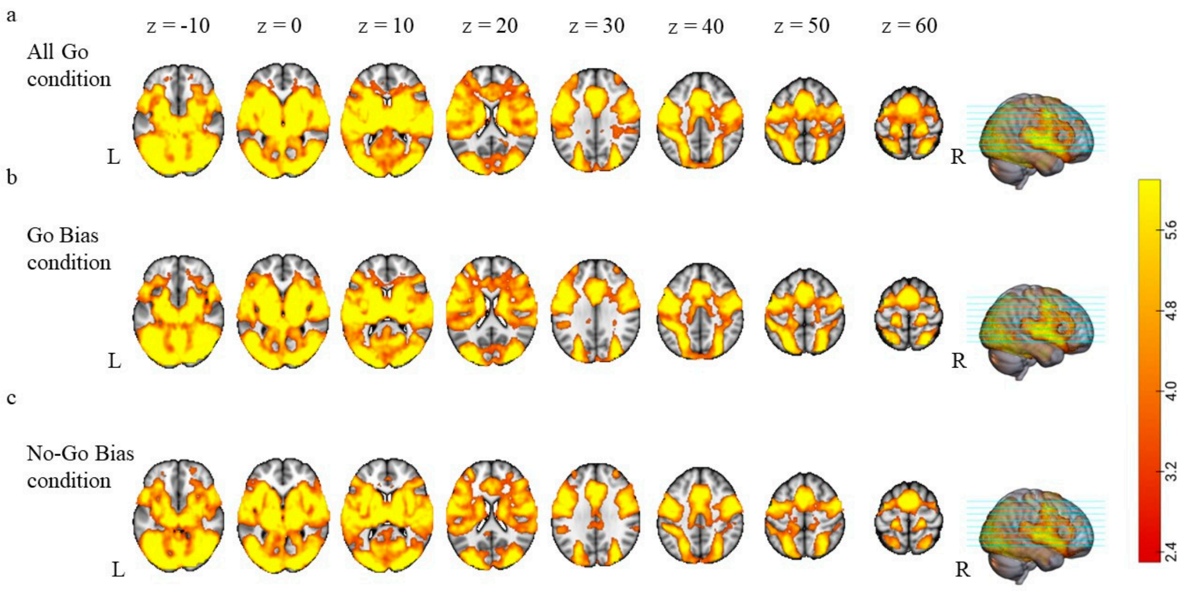

Supplementary Figure S3 *fMRI activation to the three conditions. An overview of the regions in which there was significant activation to Go trials in the (a) All Go, (b) Go Bias, and (c) No-Go Bias conditions. Slices are depicted in increments of 10 mm, starting at z = −10 and ending at z = 60. Activations were reported for clusters that had a corrected p-value of p < .05 at the cluster level.*
