# Supplementary material for: Multilingual Language Diversity Protects Native Language Production under Different Control Demands
Source: Brain Sci. 2023 Nov 13;13(11):1587. doi: 10.3390/brainsci13111587 (PMC10670415; doi:10.3390/brainsci13111587)
Supplement: Supplementary file 1 [file brainsci-13-01587-s001.zip › Table S2 Basic Activation Patterns across Three Conditions.pdf]

Supplemental Table S2 *Basic activation patterns across three conditions*

	**Hemisphere**	**Coordinates(mm)**			**AllGo**		**GoBias**		**NoGoBias**	
		**x**	**y**	**z**	**Voxels**	**Z value**	**Voxels**	**Z value**	**Voxels**	**Z value**
**Frontal pole**	**Left**	**-50**	**40**	**10**	**127372**	**5.18**	**116829**	**5.72**	**110644**	**5.70**
Frontal pole	Right	48	34	10		5.81		5.52		5.34
Paracingualte gyrus	Left	-2	32	28		5.55		5.17		6.06
Paracingualte gyrus	Right	6	32	30		5.92		4.51		5.09
Anterior Cingulate gyrus	Left	-4	30	20		5.35		4.47		5.87
Anterior Cingulate gyrus	Right	4	30	22		5.27		6.12		6.12
Frontal orbital cortex	Left	-32	30	-2		7.11		6.21		6.72
Frontal orbital cortex	Right	32	30	-2		6.37		5.87		5.86
Superior frontal gyrus	Left	-4	20	52		6.62		6.53		5.71
Superior frontal gyrus	Right	4	18	54		6.67		6.55		6.40
Insular cortex	Left	-30	16	8		7.27		6.91		7.08
Insular cortex	Right	30	16	8		7.44		6.30		6.06
Inferior frontal gyrus	Left	-46	14	6		5.86		5.86		6.42
Inferior frontal gyrus	Right	50	14	2		5.82		5.16		5.74
Middle frontal gyrus	Left	-46	6	50		5.20		5.65		4.52

Middle frontal gyrus	Right	48	6	50	5.48	5.59	5.73
Precentral gyrus	Left	-48	0	50	6.65	6.06	5.85
Precentral gyrus	Right	48	0	50	5.71	5.78	6.64
Superior temporal gyrus	Left	-64	-10	2	6.79	6.36	6.30
Superior temporal gyrus	Right	68	-20	4	7.27	6.95	6.29
Middle temporal gyrus	Left	-48	-42	4	4.32	5.20	4.68
Middle temporal gyrus	Right	54	-58	6	5.59	5.06	4.77
Postcentral gyrus	Left	-24	-34	52	4.73	5.08	4.62
Postcentral gyrus	Right	24	-32	64	6.63	5.70	5.62
Inferior temporal gyrus	Left	-54	-50	-18	6.73	7.39	6.71
Inferior temporal gyrus	Right	56	-52	-18	6.13	6.32	5.62
Ligual gyrus	Left	-16	-44	-6	5.35	5.06	5.17
Ligual gyrus	Right	24	-44	-6	6.07	5.82	5.08
Supramarginal gyrus	Left	-62	-44	16	4.95	4.22	5.08
Supramarginal gyrus	Right	66	-40	20	4.51	3.91	4.73
Angular gyrus	Left	-44	-52	12	4.26	3.60	3.13
Angular gyrus	Right	44	-52	12	4.83	4.04	3.48
Precuneous cortex	Left	-18	-60	6	5.22	5.04	5.24
Precuneous cortex	Right	24	-58	6	5.82	6.14	6.51
Lateral occipital cortex	Left	-44	-74	-14	6.49	6.55	6.52

Lateral occipital cortex	Right	44	-74	-16	6.38	6.60	6.79
Occipital pole	Left	-22	-92	-14	6.15	6.13	6.48
Occipital pole	Right	22	-94	-8	7.14	7.14	7.33
